# High and low worriers do not differ in unstimulated resting-state brain connectivity

**DOI:** 10.1038/s41598-023-28333-5

**Published:** 2023-02-21

**Authors:** Fanny Weber-Goericke, Markus Muehlhan

**Affiliations:** 1 Department of Psychology, Technische Universität Dresden, Chemnitzer Strasse 46, 01187 Dresden, Germany; 2 Department of Psychology, Faculty of Human Science, Medical School Hamburg, Am Kaiserkai 1, 20457 Hamburg, Germany; 3 ICAN Institute for Cognitive and Affective Neuroscience, Medical School Hamburg, Am Kaiserkai 1, 20457 Hamburg, Germany

**Keywords:** Prefrontal cortex, Human behaviour

## Abstract

Chronic, excessive and uncontrollable worry presents an anxiety rising and distressing mental activity relevant in a range of psychological disorders. Task based studies investigating its underlying neural mechanisms reveal fairly heterogenous results. The current study aimed to investigate pathological worry related effects on the functional neural network architecture in the resting unstimulated brain. Using resting-state functional magnetic resonance imaging (rsfMRI) we compared functional connectivity (FC) patterns between 21 high worriers and 21 low worriers. We, on the one hand, conducted a seed-to-voxel analysis based on recent meta-analytic findings and, on the other hand, implemented a data-driven multi voxel pattern analysis (MVPA) approach to yield brain clusters showing connectivity differences between the two groups. Additionally, the seed regions and MVPA were used to investigate whether whole brain connectivity is associated with momentary state worry across groups. The data did not reveal differences in resting-state FC related to pathological worry, neither by the seed-to-voxel or MVPA approach testing for differences linked to trait worry nor by using the MVPA to test for state worry related aberrations. We discuss whether the null findings in our analyses are related to spontaneous fluctuations in momentary worry and the associated presence of multiple fluctuating brain states that could cause mutually cancelling effects. For future studies investigating the neural correlates of excessive worry, we propose a direct worry induction for better control of the situation.

## Introduction

Worry presents a form of perseverative cognition^1^. It is assumed to be a part of the cognitive subdimension of anxiety^2,3^ and has been conceptualized as *a chain of thoughts and images, negatively affect-laden, and relatively uncontrollable* (Borkovec et al.^4^, p. 10). Worry can become excessive and uncontrollable. It then interferes with functioning, gets highly impairing and distressing. In this pathological form, worry represents a transdiagnostic factor, that might be involved in the onset and maintenance of several psychological disorders e.g., generalized anxiety disorder (GAD), social anxiety disorder, major depression, eating disorders^5,6^. Established cognitive models and current empirical studies state that individuals experiencing high levels of worry show attention deficits, impaired salience-processing and aberrant self-referential mental activity evident in a deficient attentional control and impoverished sustained attention, heightened sensitivity to threat and the intrusively recurring worrisome thoughts^7–11^. This results in an ongoing internal cognition even when an external focus of attention is crucial to appropriately adapt behavior. From a neurobiological view, it is assumed that such disturbances in cognitive and emotional processes result from an altered interplay between different brain networks e.g.^12,13^.

Anxiety and worry are thought to be associated with an overactive salience network (SN: see^14^ for network taxonomy) which results in an exaggerated salience detection^12,13^. In consequence, salience is misattributed to insignificant events. Regarding excessive worry it is furthermore assumed that the disturbances in the SN are accompanied by an inappropriate dynamic between the control network (CN) and the default network (DN). In response to a salient event the CN may be insufficiently engaged while the DN remains overly active due to weakened disengagement. This pattern may underlie the intrusiveness of negative thoughts, concurrent impoverished cognition and external control observed in individuals prone to engage in worrisome thoughts^15^.

Empirically, functional magnetic resonance imaging (fMRI) experiments provide some evidence that anxious worry is associated to aberrant functioning within and between those three brain networks. Studies related activation differences in central hubs of the SN, namely the anterior cingulate cortex (ACC) and the anterior insula (aI), in frontoparietal regions of the CN like the dorsolateral and the dorsomedial prefrontal cortex (dlPFC and dmPFC), and in cortical midline structures of the DN such as the middle frontal gyrus (MFG), the posterior cingulate cortex and the precuneus to pathological worry e.g.^16–20^. Considering the interaction of brain regions over time worry has been associated with increased functional connectivity (FC) between the amygdala and the dlPFC^21^, reduced FC between the ACC and the dlPFC^17^ and the dorsal aI and the dlPFC^20^. However, the pattern worry demonstrates for both activation aberrations and FC abnormalities is highly inconsistent across studies for a comprehensive review, see^22,23^.

A recent coordinate-based meta-analysis integrated the neuroimaging literature on pathological worry^23^. In this study the activation likelihood estimation method ALE^24^; was applied to determine the brain regions which most consistently show activation differences between individuals with intense and with normal worry levels. Across experiments the meta-analysis revealed convergent aberrant functioning in a left-hemispheric cluster comprising parts of the MFG, inferior frontal gyrus (IFG), and aI. These regions have been related to inhibition of unwanted, repetitive thoughts IFG^25,26^, language processes^23^, and covary with state and trait anxiety ratings^27^ left aI, left MFG^28,29^. Furthermore, structures within the derived cluster constitute important nodes within the just described brain networks: As stated above, the aI forms a key node within the SN^30^, while the MFG has been closely linked to the DN^29,31^. Functional anomalies within such nodes can be of particular significance for they may have network-wide implications and entail dysregulation within and between entire brain networks. Therefore, an important next step is to investigate the functional integration and connectivity of the derived cluster with regard to excessive worry.

The current study aims to further explore associations between pathological worry and aberrations in the intrinsic functional organization of the brain, as measured through resting-state fMRI rsfMRI^32,33^. For this purpose, we applied a hypothesis- as well as a data-driven approach. Building on the cluster identified in the meta-analysis described above, which includes parts of the MFG, IFG, and aI, we conducted a seed-based hypothesis-driven analysis to test for differences in the pattern of resting-state FC (rsFC) between high worriers (HW) and individuals with normal worry levels (low worriers, LW). Given the wide variability of FC abnormalities related to worry, that have mainly been determined by seed-based analyses e.g.^17,20,34^, we furthermore exploited a hypothesis-free, data-driven multi-voxel pattern analysis (MVPA) aimed to identify brain regions with divergent FC patterns between HW and LW. The thus obtained regions can then be used as seed regions in post-hoc seed-to-voxel analyses to discover how specifically the connectivity between these brain areas and the rest of the brain is tied to trait worry tendency.

Moreover, this study exploratively investigates whether differences in rsfMRI connectivity correlate with levels of state worry across the experimental groups. In contrast to other anxiety symptoms, the occurrence of worry is relatively stimulus independent, which means that worry might arise without environmental input^35,36^. Consequently, it is likely that participants in a given HW sample differ in their current mental state during a given time of measurement. Accordingly, the detectable abnormal brain configurations might differ among participants within a HW sample in dependence on state worry. It is therefore reasonable to consider momentary assessments of the worry state in addition to trait worry^37^. For this reason, we examined, on the one hand, whether self-reported state-worry levels are associated with the connectivity pattern of the seed regions described above. On the other hand, we applied a MVPA, to identify brain regions where connectivity values vary as a function of state worry. Identified regions can subsequently serve as seed regions for network architecture analyses.

## Materials and methods

### Participants and procedure

The sample for this study comprised 42 participants (4 men, age: 29.3 (5.2), 38 women age: 26.2 (6.2)). All participants were right-handed and had normal or corrected to normal vision. General exclusion criteria were: presence or history of bipolar, borderline-personality, psychotic disorder, substance abuse, seizures or head injury, suspected or confirmed pregnancy and lacking MRI compatibility (i.e. magnetic or electronic implants). Participants were recruited from direct local advertisement through fliers, and health insurance journals, as well as from the research registry of the Neuroimaging Centre Technische Universität Dresden.

Participants initially completed an online-screening which contained the Penn State Worry Questionnaire PSWQ^38^; an instrument designed to provide a trait assessment of pathological worry. Based on their total PSWQ score participants were then assigned to either the HW group (PSWQ ≥ 56; people who experience excessive, chronic and uncontrollable worry) or the LW group PSWQ ≤ 44; classification into groups was based on the cut-offs established in the literature to reliably distinguish between pathological and normal worry levels^39,40^. To further prove eligibility participants were invited to a personal screening appointment, which consisted of an in-house checklist on chronic physical diseases and medication. Moreover, to confirm the absence of any current or past mental disorder in the healthy control group and to grant comprehensive diagnosis of mental disorders in the HW group the two-phase design of the Munich Composite International Diagnostic Interview (M-CIDI; Wittchen and Pfister^41^) was used. First, all participants were screened for psychological disorders using the Stamm-Screening Questionnaire (SSQ; Wittchen and Pfister^41^). This screening inventory contains 16 items to assess the major mental disorders and each item represents an entry question to a corresponding section of the structured clinical interview M-CIDI. If such an entry question is answered positively, then the corresponding section of the M-CIDI is used to verify the actual current or lifetime presence of the mental disorder indicated by the screening. For all participants in the LW group, screening was negative. With those participants in the HW group with positive screenings, the structured clinical interview M-CIDI was conducted in the second step for all sections for which the screening was positive to assess the presence of mental disorders on the basis of DSM-IV-TR criteria^42^. For the scanning session, participants were scheduled for a second appointment at the Neuroimaging Centre Dresden. All participants completed the PSWQ again at the scanning appointment. Only participants who met the PSWQ cut-offs for the respective group at the time of fMRI testing were included in the analyses, resulting in 21 participants per group.

M-CIDI indicated that 10 HW participants met DSM-IV criteria for at least one mental disorder. Current axis-I conditions included GAD (*n* = 3), social anxiety disorder (SAD, *n* = 3), major depression (recurrent, *n* = 1), specific phobia (blood-injection-injury type, *n* = 1; situational type, *n* = 1), posttraumatic stress disorder (PTSD, *n* = 3), undifferentiated somatoform disorder (*n* = 3), somatoform pain disorder (*n* = 1) and bulimia nervosa (*n* = 1). Four participants fulfilled diagnostic criteria for more than one disorder. Three of them were comorbid with two (GAD and SAD; GAD and PTSD; undifferentiated somatoform disorder and bulimia nervosa) and one with four diagnoses (GAD, PTSD, SAD and specific phobia, situational type).

At the time of testing one participant of the HW group was taking regular antidepressants and amphetamines (Citalopram and Medikinet) at a stable dose. None of the remaining participants experiencing high levels of worry were under pharmacological or psychotherapeutic treatment. Also, all participants in the LW group were free of any psychiatric medication. The study was approved by the Ethics Committee of the Technische Universität Dresden [EK: 10012012]; all participants provided written informed consent and received an expense allowance for participating.

### fMRI data acquisition

In the MRI scanner participants underwent an anatomical scan and four blocks of an emotion-regulation task (as part of a larger fMRI study), followed by the 7-min resting-state scan, in which they were told to keep their eyes open and to rest. In total the MRI session took 69 min. To obtain MRI images a 3 Tesla Trio Tim Siemens MRI scanner (Siemens Healthcare GmbH, Erlangen, Germany), equipped with a 12 channel head coil, was used. For anatomical reference structural (T1-weighted) images were acquired using a Magnetization Prepared Rapid Gradient Echo Imaging (MPRAGE) sequence (176 sagittal slices; slice thickness 1 mm; TR 1900 ms; TE 2.26 ms; flip angle 9°; field of view (FOV) 256 × 256 mm; matrix size 256 × 256). During the resting-state measurement, functional (T2*-weighted) images were obtained in an ascending order using an echo planar imaging (EPI) sequence with 42 axial slices (slice thickness 2 mm) per volume (TR 2410 ms; TE 25 ms; flip angle 80°; slice gap 1 mm; FOV 192 × 192 mm; matrix size 64 × 64).

### Self-report assessments and state worry assessment

In addition to the PSWQ all participants completed the Generalized Anxiety Disorder Questionnaire-IV GAD-Q-IV^43^ and the Beck Depression Inventory-II BDI-II^44^.

Immediately after completion of the resting-state scan, levels of state worry occurring during the resting-state measurement were assessed by participants’ ratings. Participants were asked to rate on three separate visual analog scales (VASs, coded from 0 to 4) the extent, intensity and frequency with which they had experienced worrisome thoughts for the duration of the preceding resting period (see Supplemental Material A for explicit wording). Participants who negated any worrisome thoughts with their response to the first question were not asked for intensity and frequency. In the analyses this data was substituted by 0. The intensity and frequency with which worry is experienced are crucial factors for the degree of impairment caused by worry^45,46^. They are therefore critical for the evaluation of the clinical relevance of worry. For this reason, we multiplied frequency and intensity ratings of each subject to form a state worry index that is meaningful in terms of its clinical relevance.

### Statistical analyses

#### Analysis of sociodemographic data, clinical questionnaires and rating data

Analyses of sociodemographic data, clinical questionnaires, and rating data were performed using Stata 15.1^47^. Differences at *p* < 0.01 are regarded as significant. To test for group differences, *t* tests, Fisher’s exact tests and Wilcoxon rank-sum tests were conducted on self-report sociodemographic, clinical and state worry measures.

#### fMRI data analysis

##### Preprocessing

FMRI data were preprocessed and analyzed using the CONN-toolbox v.20b^48^ running in Matlab R2018b (The MathWorks Inc., Natick, MA, USA). The first 4 volumes of the resting-state run were discarded prior to preprocessing to allow for T1 equilibration effects. The remaining 193 scans were corrected for slice time errors, spatially realigned and unwarped to correct for head movement artifacts. Furthermore, functional images were detected for outliers (ART-based scrubbing). Structural and functional images were segmented and directly normalized to the MNI (Montreal Neurological Institute, Quebec, Canada) reference brain. Subsequently, functional scans were spatially smoothed (8 mm full-width half maximum [FWHM] Gaussian kernel). These steps are implemented by default in the CONN toolbox preprocessing pipeline and based on SPM 12 (Wellcome Department of Imaging Neuroscience, UCL, London, UK). We then proceeded with the denoising step in CONN including detrending and the component-based correction method (CompCor; Behzadi et al.^49^) and band-pass filtering of 0.008–0.09 Hz to remove subject-specific movement and physiological noise factors as well as noise regions of interest (ROIs; white matter and cerebrospinal fluid time series). In addition, six head movement parameters and the ART-detected outliers were included as nuisance regressors on first-level analyses.

##### Seed-based connectivity analyses

**Figure 1:**
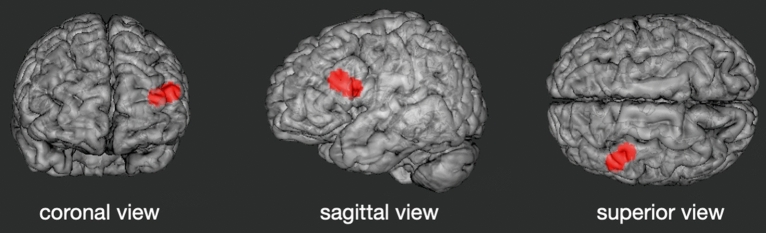
Location of the ROI mask representing the left-hemispheric ALE-cluster used in the seed-based connectivity analyses.

As stated in the introduction, the seed regions were selected on the basis of recent meta-analytical findings of brain locations that consistently show activation differences between HW and LW across various studies^23^. This ALE-meta-analysis revealed a left-hemispheric cluster covering parts of the MFG, IFG and aI cortex. Within this cluster the ALE analysis identified two peak voxels (located in the MFG and insula) specifying the precise locations with the most agreement across studies. The coordinates of these two peak voxels were used in the current study to create the seed region. Masks were generated using the WFU PickAtlas tool^50,51^. Therefore, 6 mm spherical 3D volumes were built around the peak-voxels’ coordinates [− 46, 24, 16 and − 40, 14, 12] of the ALE-cluster. Further calculations were performed with the resultant spheres as separate seed regions as well as with them combined to one mask to model the full ALE-cluster (Fig. 1 illustrates the location of the seed regions). The Conn toolbox extracts the average BOLD time series across all the voxels within the given ROI. Then, seed-to-voxel connectivity maps for each subject were calculated. Thereafter, these measures were entered into a second-level general linear model to compare FC patterns between the HW and the LW group. Age and sex were entered as covariates of no interest. The voxel-level height threshold was set at *p* < 0.001 uncorrected and the cluster-level extent threshold at *p* < 0.05, familywise error-corrected (FWE) for multiple comparisons.

In addition to comparing the functional connectivity between the two groups, we exploratively analyzed whether the seed-based connectivity correlates with the state worry index across all participants. Results of this second-level analysis were thresholded at a height threshold of *p* < 0.001 uncorrected and a cluster-mass *p*-FWE < 0.05 threshold applying nonparametric statistics.

##### Multi-voxel pattern analysis (MVPA)

To identify further optimal seed regions for brain connectivity analyses in relation to both trait and state worry we calculated a multi-voxel pattern analysis (MVPA), a data-driven approach implemented in the CONN toolbox. This method has been used in several recent studies e.g.^52–54^. MVPA is a voxel-to-voxel measure which yields the connectivity pattern between each voxel of the brain and the remaining brain voxels using a few abstract components. On the first-level for each voxel within the gray matter template we computed the FC to the rest of the brain (i.e., all other brain voxels). The dimensionality of the resulting multi-voxel pattern was reduced by performing a principal component analysis (PCA), which aims to maximize the between-subject variability in the resulting patterns with a lower number of spatial components^54^. As defined by default, we retained 8 components to maintain approximately a 1:5 ratio between the number of components extracted and the number of participants. At second-level, we performed two multivariate pattern analyses: the first one to test for rsfMRI connectivity differences associated with trait worry looking for significant differences in the resulting principal component scores between the HW and the LW group. The second one to test for associations between any of the 8 component scores for each subject and self-report measures of state worry during rsfMRI. Age and sex were used as nuisance regressors in both analyses. We used nonparametric statistics and applied a voxel-wise height threshold of *p* < 0.01, uncorrected and a cluster-mass threshold at a false discovery rate (FDR) corrected significance level of *p* < 0.05. This second-level statistical analysis reveals voxel clusters that exhibit FC patterns to the rest of the brain which are modulated by the regressor (i.e. trait and state worry). Since the MVPA is an omnibus test, post-hoc analyses using the resultant significant clusters as ROIs in seed-to-voxel analyses need to be conducted to characterize the identified connectivity differences procedure as described in other research studies, e.g.^54,55^.

### Ethics approval

The study was approved by the Ethics Committee of the Technische Universität Dresden [EK: 10012012]. The procedures used in this study adhere to the tenets of the Declaration of Helsinki.

### Consent to participate

All participants provided written informed consent to participate in the study.

## Results

### Self-report measures

Descriptive statistics and self-report measures are presented in Table 1. Groups did not differ in age, sex and educational level. The groups did show statistically significant differences with regard to clinical questionnaires: as expected, the HW group reported higher levels of dispositional worry than the LW group (PSWQ). In addition, 16 of the 21 participants in the HW group achieved a GAD-Q-IV score above the cut-off indicating the presence of a GAD diagnosis, whereas this was not the case in any of the LW participants. Also, the HW group reported mild-to-moderate levels of depressive symptoms, while the LW group's scores remained in the non-clinical range (BDI-II). Besides that, a main effect of group was evident for levels of state worry, with the HW group engaging in worrisome thinking more than the LW group during the rsfMRI measurement (Supplemental Material Fig. B.1 illustrates the distribution of state worry per group, Supplemental Material Table B.2 lists the number of observations by values of the state worry index variable per group).

**Table 1:** Subjects’ sociodemographic, clinical and state worry data.

	LW (*n* = 21)	HW (*n* = 21)	Statistics *p*
Sex (M/F)	3/18	1/20	0.29
Age (years), Mean (S.D.)	25.29 (4.00)	27.62 (7.65)	0.22
Educational level, n (%)			0.39
Low	4 (19)	3 (14.3)	
Medium	13 (61.9)	10 (47.6)	
High	4 (19)	8 (38.1)	
PSWQ, Mean (S.D.)	32.43 (5.68)	64.14 (5.21)	< 0.001
GAD-Q-IV, (subjects above cut-off*) / Mean (S.D.)	0/21.54 (.55)	16/218.28 (2.87)	< 0.001
BDI-II, Mean (S.D.)	1.45 (1.64)	16.81 (6.86)	< 0.001
State worry rating: extent, Median (IQR)	0.02 (.75)	1.85 (0.63)	< 0.001
State worry rating: frequency, Median (IQR)	0 (0)	1.37 (0.66)	< 0.01
State worry rating: intensity, Median (IQR)	0 (0)	1.52 (1.02)	< 0.01
State worry rating index: intensitiy*frequency, Median (IQR)	0 (0)	2.26 (2.82)	< 0.01

*BDI-II* beck depression, *F* female, *GAD-Q-IV* generalized anxiety disorder questionnaire-IV, *HW* high worriers, *IQR* interquartile range, *LW* low worriers, *M* male, *PSWQ* penn state worry questionnaire, *S.D.* standard deviation.

*A score above the cut-off is indicative of a GAD diagnosis.

### fMRI results

#### Seed-based connectivity analyses

The seed-to-voxel analyses using the mask modeling the MFG-IFG-aI cluster and the separate masks built around the two ALE peak voxel coordinates as seed regions (identified by prior ALE meta-analysis) did not reveal differences in connectivity patterns between the HW and the LW group. Likewise, the exploratory analyses showed no correlation between the ALE-cluster connectivity and the state worry index.

#### MVPA

The MVPA revealed neither brain clusters with different FC patterns between the HW and the LW group, nor brain clusters whose FC patterns significantly correlated with levels of state worry.

## Discussion

The current study investigated whether pathological worry is associated with differences in resting-state functional brain connectivity. To this end, we selected seed brain regions based on recent meta-analytic findings and compared their rsFC patterns between HW and LW and tested them for correlations with state worry. In addition, we applied a data-driven MVPA approach on the rsfMRI data to identify brain areas with FC patterns associated to trait worry on the one hand and to state worry on the other. However, our data did not reveal any worry related differences in rsFC neither by the categorical separation into HW and LW based on dispositional worry tendency nor by testing on momentary state worry levels.

On group level we examined the rsFC pattern of a brain region covering parts of the aI, the MFG and the IFG which has meta-analytically been associated to pathological worry^23^. We did not detect any rsFC differences between the aI-MFG-IFG cluster and the rest of the brain between HW and LW. The core difference between our study and the studies entering the meta-analysis is the presence of external emotional cues. All studies integrated in the meta-analysis looked at functional differences in the brains of HW when actively processing external emotional stimuli e.g.^56–59^. In contrast participants in our study were resting in the scanner, not given any external emotional input. If the functional differences identified in the meta-analysis reflected aberrant salience processing in response to external stimulation, no FC differences of the corresponding brain areas in the unstimulated resting brain might be explainable: without external stimulation no saliency detection and mapping takes place. If, however, such functional differences were associated with aberrant inner speech processes in HW, they should also arise when worry occurs spontaneously. In this case, the chosen seed-based rsFC analysis might have been too restrictive in terms of time and region to detect such fluctuations of spontaneous worry.

Moreover, the data-driven MVPA approach also did not reveal differences in the intrinsic functional network architecture of the resting brain associated to dispositional worry. It is possible that this null-result is also related to the characteristic of worry that it has the potential to arise spontaneously and without external input e.g.^36^. Fonzo and Etkin^35^ recently proposed a model to explain the variable pattern of activation and connectivity abnormalities found in task-based imaging studies of pathological worry. They suggest that compared to healthy controls individuals with GAD display a greater variability of different brain states. The model proposes that depending on the current internal degree of worry various patterns of engagement or disengagement of brain regions involved in emotion reactivity and emotion regulation may evolve. Moreover, shifting among the various brain states is supposed to take part in an inflexible relatively stimulus-independent manner. According to this model the presence as well as the direction of rsFC abnormalities depends on which of the potential brain configurations are present in the given sample at the time of measurement. If variance across such brain states in a given HW sample is high mutually offsetting effects might be at play and FC abnormalities might not be detected. As participants’ ratings on their internal worry state indicate that might have been the case in our HW sample: one third of our HW sample reported no worry, approximately another third reported very mild worry and ratings of the final third were distributed between medium and high worry levels during the rsfMRI period. Unfortunately, the small sample size did not allow building further sub groups based on the state worry degree.

However, we tested whether rsFC correlated with state worry levels across groups by examining the correlations to the rsFC patterns of our seed region (the aI-MFG-IFG cluster) and also by applying the MVPA approach. HW reported higher engagement in worrisome thoughts during the resting-state measurement than LW. Though, the FC patterns of our seed region did not correlate with state worry and the rsfMRI data did not reveal brain clusters where connectivity patterns covaried with self-reported worry state levels across groups. We assessed state worry by VASs, which might not have depicted state worry levels sufficiently fine enough over the time course of the resting-state period.

To date only a few studies have used comparable VASs to investigate momentary worry levels in the scanner and found state worry related effects in the fMRI-signal. Some differences exist between the current study and these previous studies. Prior studies have used some form of intervention e.g., worry induction^60,61^, while the present study assessed spontaneously occurring worry during a stimulus free resting-state period to investigate its neural correlates. In consequence, the overall occurrence of spontaneous worry in our sample might have been too low to detect related FC patterns. Furthermore, we assessed state worry retrospectively for a 7-min resting-state period. In contrast Servaas et al.^61^ asked participants to rate their worry intensity for a time period of 15 s following neutral or worry inducing sentences. And Macovac et al.^62^ used a low demand tracking task as an objective indicator of attentional shifts by decreasing or increasing reaction times. Whenever reaction time changes were detected, participants were presented the VASs to measure the presence of perseverative cognition. Thus, VASs as such appear to work, and the application of VASs to assess state worry related brain network differences might benefit from (1) worry inductions to stimulate a broader range of worry intensities and (2) a higher temporal resolution for a more accurate assessment of state worry fluctuations.

Several limitations of the study design should be mentioned that may have contributed to the null findings. First, in the current study pathological worry was investigated as a transdiagnostic factor in line with the Research Domain Criteria initiative^63^. Consequently, our HW sample was selected based on high levels of trait worry rather than on clinical diagnosis. The HW sample included individuals who met diagnostic criteria for GAD or other mental disorders as well as individuals who did not meet criteria for a psychological disorder (see Section “Participants and procedure” for details). The heterogeneity regarding psychopathology in our sample might have covered worry-related effects. With the research regarding the neuronal circuits underlying pathological worry still in its infancies it might be advisable to focus on samples who report worry as the primary psychopathological symptom (e.g. GAD or HW without psychological diagnoses except for GAD). Worry, however, is constituted as a transdiagnostic construct, and empirical findings suggest that the worry we observe among different psychopathologies, in individuals who worry excessively but do not meet diagnostic criteria for a psychiatric disorder, and in individuals with normal worry, is quantitatively rather than qualitatively different e.g.^40^. Therefore, it might be more accurate to follow a continuous approach rather than to form groups based on cut-off scores of continuous worry measures or on GAD diagnostic criteria. Such an approach, which accounts for the full spectrum of worry severity, would result in an increase in statistical power and thus may be more appropriate for investigating worry independent of primary psychopathology. To separate effects related to worry from those related to psychopathology, the sample size should ideally also allow continuous analyses to be combined with group comparisons based on the severity of worry and the presence of psychopathology. This would lead to the formation of three groups: healthy LW, healthy HW and HW with psychopathology.

Second, participants in our sample are predominantly female. Given this sex distribution, the present null results should be interpreted mainly for females and call for an explicit investigation in male-only samples.

Third, the resting scan sequence was performed after an emotion regulation task in which all participants were presented with negative and neutral images from the international affective imagery system. This order in the design might have been unfortunate because it possibly modified the resting-state results. Given that worry is triggered by threat representations, it is likely that the confrontation with negative images in HW leads to bouts of worry, that sustain and are difficult to control. This may have led to differential engagement in worry state within the HW group that persisted during the resting-state measurement. Repeated confrontation with negative images may also induce a more alert, anxiously aroused state in LW compared to their normal baseline state, possibly associated with a neural pattern more similar to that of individuals with high worry proneness. Such effects of the preceding emotion regulation task may have reduced the likelihood of detecting group differences in the FC patterns in the resting brain between high and low worriers.

Fourth, the sample size of 21 participants per group may have resulted in too little statistical power to detect small effects. Furthermore, the sample size also set a limit for conducting sub-analyses to examine effects of different state worry levels or psychopathologies in the HW group.

## Conclusion

Using rsFC analysis methods, the current investigation did not find differences in spontaneous brain activity between HW and LW or related to state worry. The authors assume that the null findings presented here do not necessarily indicate the absence of connectivity-based differences in the resting, unstimulated brain between individuals with excessive worry proneness and healthy individuals with normal worry. We reported several factors that may have contributed to the null results, such as the formation of experimental groups based on cut-off scores of continuous worry measures, or use of overly restrictive analytical methods to capture state worry related FC changes. Moreover, other empirical studies have shown that rsFC abnormalities are characteristic of excessive worry^21,64,65^. However, the overall heterogeneity in terms of localization, direction and presence of abnormalities in rsFC studies including the present one might relate to fluctuations of different mental states that have been suggested to be inherent to pathological worry^35^. Therefore, other analytical methods that capture how rsFC patterns change over time may be better suited to study worry e.g. dynamic rsFC analysis^66,67^; and may yield novel insights into the abnormal functional organization of the brain in excessive worriers.

## Data availability

The datasets generated during and/or analysed during the current study are available from the corresponding author on reasonable request.

## Supplementary Information

Supplementary Information.
