# Supplementary material for: High and low worriers do not differ in unstimulated resting-state brain connectivity
Source: Sci Rep. 2023 Feb 21;13:3052. doi: 10.1038/s41598-023-28333-5 (PMC9944913; doi:10.1038/s41598-023-28333-5)
Supplement: Supplementary file 1 — Supplementary Information. [file 41598_2023_28333_MOESM1_ESM.pdf]

Supplemental Material

High and low worriers do not differ in unstimulated resting-state brain connectivity

Fanny Weber-Goericke[a], Markus Muehlhan[b,c]

[a] Department of Psychology, Technische Universität Dresden, Chemnitzer Strasse 46, 01187 Dresden, Germany

[b] Department of Psychology, Faculty of Human Science, Medical School Hamburg, Am Kaiserkai 1, 20457 Hamburg, Germany

[c] ICAN Institute for Cognitive and Affective Neuroscience, Medical School Hamburg, Am Kaiserkai 1, 20457 Hamburg, Germany

Correspondence: fanny.weber-goericke@tu-dresden.de; markus.muehlhan@medicalschool-hamburg.de

Supplemental Material A: State worry assessment

For the assessment of state worry participants were initially asked: "To what extend did you worry about different topics?". They responded by horizontally moving a slider across the visual analog scale which was subdivided into four parts (not at all – little – moderate – a lot). Participants who indicated to have experienced worry to some extent were further asked "How intense were these worries?" and "How frequent were these worries?". They again answered by moving the slider, this time to a position between the poles "not very intense" and "very intense", and "very rarely" and "very frequent", respectively.

Supplemental Material Fig. B.1: Distribution of self-reported state worry levels in the low and high worrier group

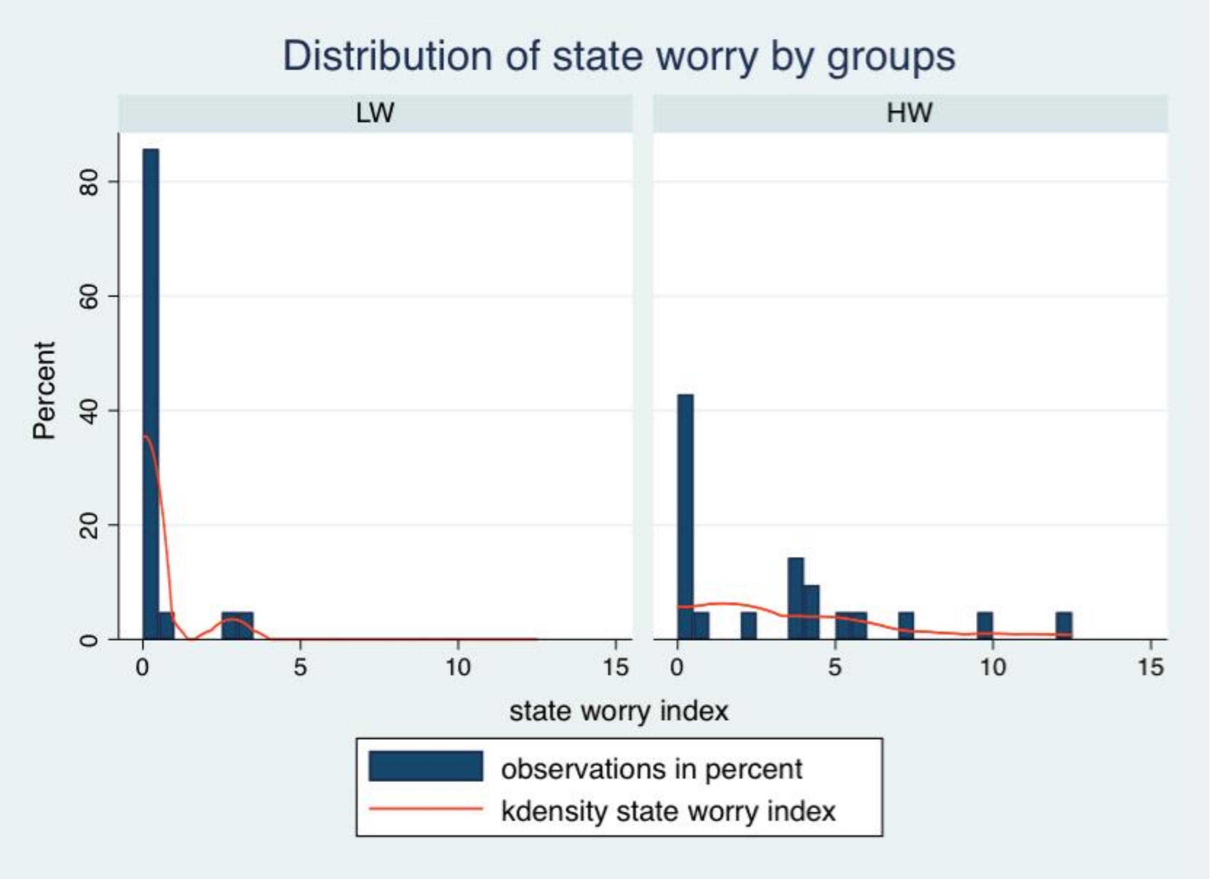

Fig. B.1: Distribution of self-reported state worry levels. The graphs show the distribution of the variable state worry index by group.

Supplemental Material Table B.2: Number of observations by values of the state worry index variable per group.

state worry index	Freq	Percent	Cum
HW			
0	7	33.33	33.33
.34	1	4.76	38.10
.25	1	4.76	42.86
.93	1	4.76	47.62
2.4	1	4.76	52.38
3.53	1	4.76	57.14
3.64	1	4.76	61.90
3.88	1	4.76	66.67
4	1	4.76	71.43
4.06	1	4.76	76.19
5.08	1	4.76	80.95
5.82	1	4.76	85.71
7.39	1	4.76	90.48
9.95	1	4.76	95.24
12.35	1	4.76	100.00
Total	21	100.00	
LW			
0	16	76.19	76.19
.17	1	4.76	80.95
.48	1	4.76	85.71
.50	1	4.76	90.48
2.56	1	4.76	95.24
3.09	1	4.76	100.00
Total	21	100.00	

Cum, cumulative; Freq, frequency; HW, high worriers; LW, low worriers.
